# A simple way to distinguish bed clothing contamination in a whole body bone scan: a case report

**DOI:** 10.1186/1752-1947-1-173

**Published:** 2007-12-05

**Authors:** Majid Assadi, Abdolali Ebrahimi, Mohammad Eftekhari, Armaghan Fard-Esfahani, Mojgan Nazar Ahari, Iraj Nabipour, Farshid Gheisari, Shabnam Shahbaz, Reza Baghaei, Sakineh Assadi

**Affiliations:** 1 Department of Nuclear Medicine and Oncology, The Persian Gulf Health Research Center, Busheher University of Medical Sciences, Bushehr, Iran; 2 Research Institute for Nuclear Medicine, Shariati Hospital, Tehran University of Medical Sciences, Tehran, Iran; 3 Nuclear medicine department, Namazi Hospital, Shiraz University of Medical Sciences, Shiraz, Iran

## Abstract

Whole body bone scan with Technetium-99m MDP (methylene diphosphate) can detect bony lesions due to altered osteoblastic activity.

Non-physiologic or increased radiotracer uptake in the bony structures of patients with a history of malignant diseases is usually interpreted as being suspicious of bone metastasis. It is extremely important to properly distinguish false positive sites of Tc-99m MDP localization. We present three patients with the same pattern of Tc-99m MDP abnormality in different locations. These scans were all performed on the same day to evaluate possible bone metastases in three patients with breast carcinoma. After careful examination, repeated images revealed bed clothing contamination. This is different from bed contamination by displacement among different patients. It is also different from detector contamination by limited area of involvement where detector contamination appears as a line throughout the total body projection. It can be helpful if a nuclear medicine specialist has a brief look at all scans prior to reporting them. In cases where the same pattern of abnormality is repeated in all images, the possibility of technical error such as bed clothing contamination rather than a pathological abnormality should be borne in mind.

## Introduction

Whole body imaging with Tc-99m MDP is able to detect bone metastasis. After administration of Tc-99m MDP physiologic activity is noted in a fairly homogeneous pattern in bony structures, and this is generally symmetrical. Urinary bladder and kidneys are visualized as normal findings [1]. However, on some occasions, increased activity in the bony or extra-skeletal structures is not due to bone-related pathologies. Contamination is one of the most important causes [1]. We describe three patients with the same pattern of Tc-99m MDP contamination occurring in different places. All of the scans were performed on a same day in patients to exclude possible bone metastasis from breast carcinoma.

## Case Presentation

### Case-1

**Figure 1:**
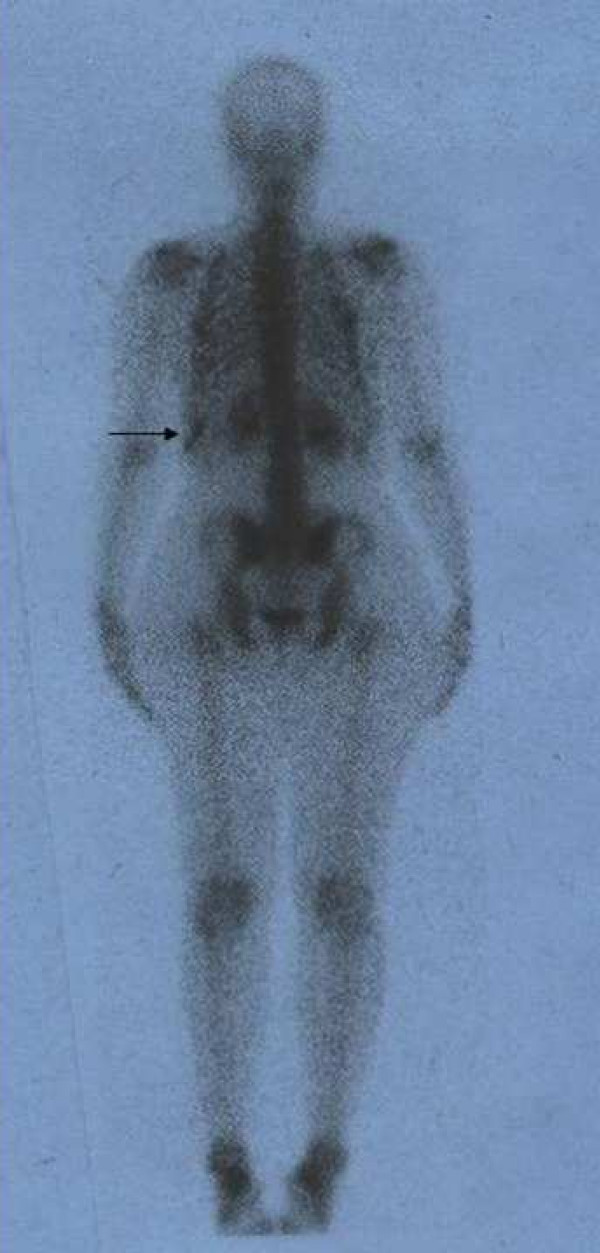
Posterior whole body bone scan shows linear increased activity at the region of the left lower ribs due to bed clothing contamination.

A 35 year old woman was referred to our center for a whole body bone scan to exclude possible bone metastasis. She had breast cancer and had undergone right mastectomy 17 days ago. She had received no radiotherapy or chemotherapy and she had no complaints of bone pain. The posterior image showed a linear activity in the left lower chest (fig 1). Careful observation showed no abnormality on the anterior projection. The possibility of contamination was considered. After changing her bed clothing, the abnormal tracer uptake disappeared.

### Case-2

**Figure 2:**
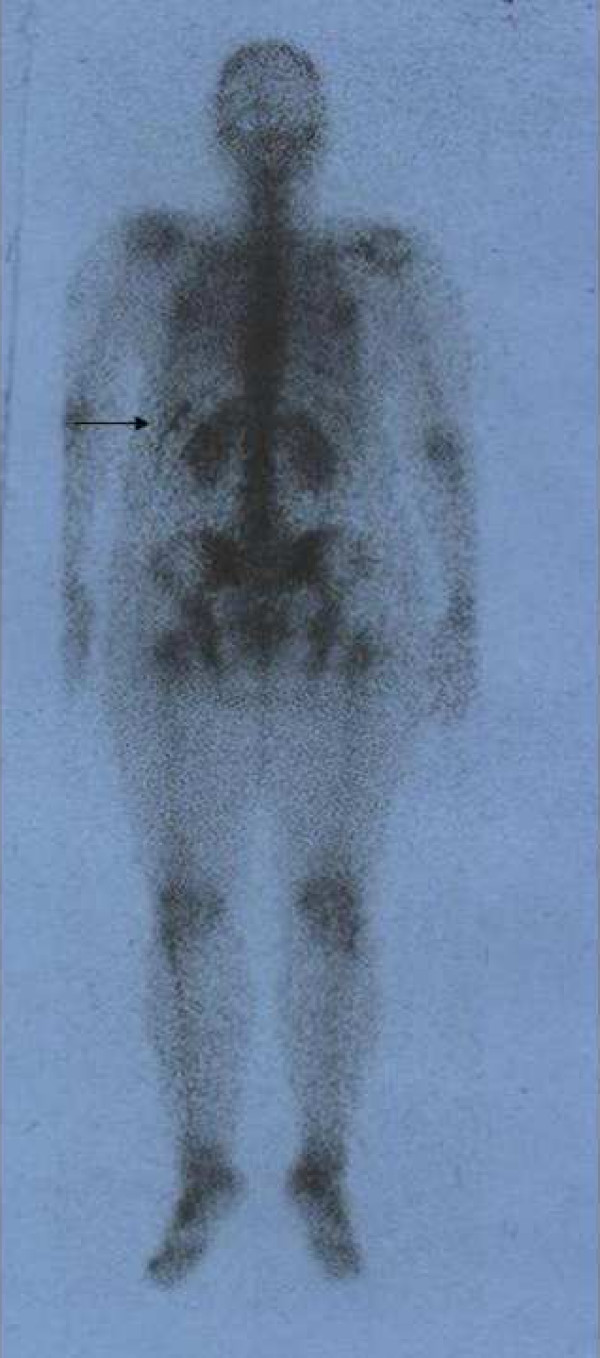
Whole body bone scan on posterior view depicts a linear pattern of increased tracer accumulation at the level of the left lower ribs due to bed clothing contamination.

A 45 year old woman, with a history of adenocarcinoma of the right breast and a mastectomy performed two years ago, was referred for whole body bone scan. Again there was a linear pattern of activity in the posterior view of the left lower chest (fig 2). The image showed no activity in that region after changing the bed clothing.

### Case-3

**Figure 3:**
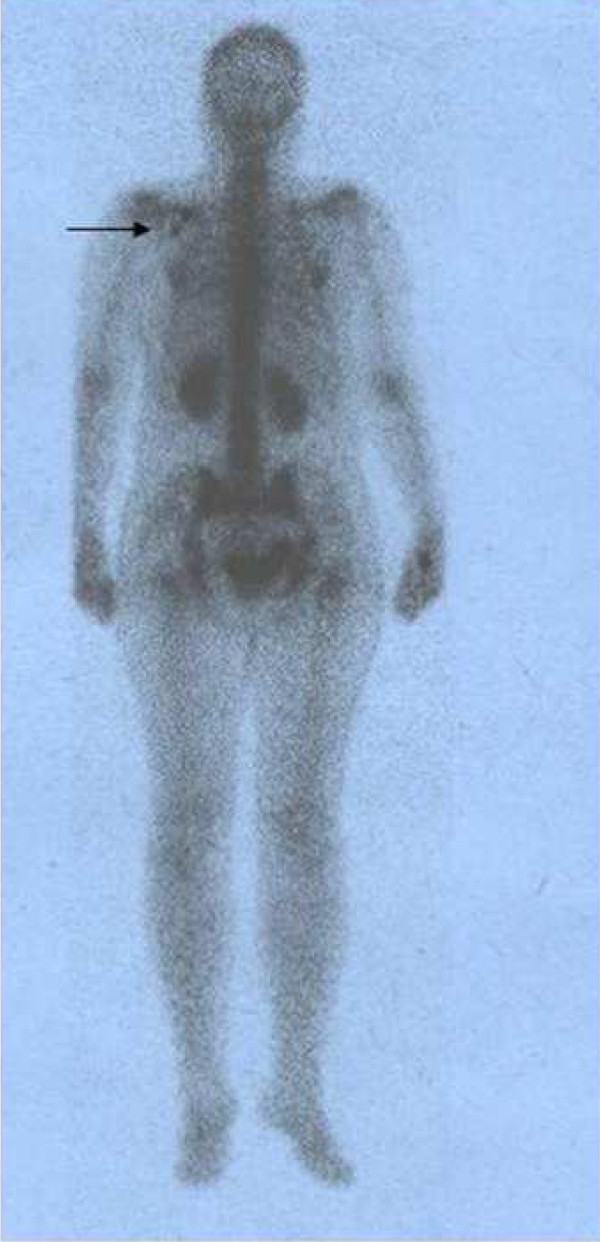
This figure shows linear activity in the left shoulder region which is due to bed clothing contamination and which resolved after removing contaminated bed clothes.

A 43 year old woman, who had undergone left mastectomy four years ago, was referred to our center for a follow-up bone scan. On posterior projection there was abnormal activity with linear pattern in the left shoulder region (fig 3). After removing the bed clothing, the focal activity was not visualized in repeat images.

## Discussion

Tc-99m MDP whole body scan is a valuable procedure for detecting bone metastasis; however the possibility of false positive results due to technical error or contamination should always be kept in mind. This is particularly true for lesions seen only on a single view [2]. A common source of contamination results from the intravenous injection of the radioisotope. Contamination may result from injection site dose infiltration, a leaking intravenous tube, or from bleeding from the puncture site because of inadequate hemostasis [3]. Also, urinary contamination is a relatively common finding in whole body bone scan (WBBS) [3].

Sometimes the body or clothing contaminants migrate through perspiration-soaked areas, typically the knees and forearms, contaminating a protective cloth or table, which may result in confounded and misinterpreted final images [2]. Some reports also indicate adverse working conditions, including hot and humid or cramped working environments, can cause contamination [2]. There are several methods to control these factors. For example, decontaminating different anatomical sites on the body, to eliminate or reduce the uptake of radioactivity absorbed into the body, and cleaning and decontaminating dry and wet surface, equipment and clothing.

We observed a similar pattern of abnormality in different places in three referred patients. We considered the possibility of technical error as an explanation for these findings. After careful examination and multiple spot views, bed clothing contamination was confirmed as the cause of these abnormalities. Clothing contamination differs from bed contamination due to the observation of changeable locations in different patients. It is also different from detector contamination by its limited area of involvement since detector contamination appears as a line throughout the whole body on the total body projection [2].

## Conclusion

Our findings suggest that if on brief review of images performed on a same day, the same abnormal pattern of activity is found in different bone scans, technical errors or contaminations rather than actual bone abnormalities should be the first consideration.

## List of abbreviations used

Methylene diphosphate (MDP), whole body bone scan (WBBS)

## Competing interests

The author(s) declare that they have no competing interests.

## Authors' contributions

MA participated in its design and coordination and helped to draft the manuscript and provided interpretation of the scintigraphic figures. AE revised the article for intellectual content details and helped to draft the manuscript. ME, AFE, MNA, IN, FG, S S, RB, SA supervised the acquisition process and interpreted the scintigraphic images. All authors read and approved the final manuscript.

## Consent

Written informed consent was obtained from all three patients for publication of this case report and any accompanying images. Copies of these written consents are available for review by the Editor-in-Chief of this journal.
